# Identifying patterns in foraging-area origins in breeding aggregations of migratory species: Loggerhead turtles in the Northwest Atlantic

**DOI:** 10.1371/journal.pone.0231325

**Published:** 2020-04-13

**Authors:** Joseph B. Pfaller, Mariela Pajuelo, Hannah B. Vander Zanden, Kimberly M. Andrews, Mark G. Dodd, Matthew H. Godfrey, DuBose B. Griffin, Breanna L. Ondich, S. Michelle Pate, Kristina L. Williams, Brian M. Shamblin, Campbell J. Nairn, Alan B. Bolten, Karen A. Bjorndal

**Affiliations:** 1 Caretta Research Project, Savannah, Georgia, United States of America; 2 Archie Carr Center for Sea Turtle Research and Department of Biology, University of Florida, Gainesville, Florida, United States of America; 3 Pro Delphinus, Lima, Peru; 4 Georgia Sea Turtle Center, Jekyll Island, Georgia, United States of America; 5 Odum School of Ecology, University of Georgia, Athens, Georgia, United States of America; 6 Georgia Department of Natural Resources, Brunswick, Georgia, United States of America; 7 North Carolina Wildlife Resources Commission, Beaufort, North Carolina, United States of America; 8 South Carolina Department of Natural Resources, Charleston, South Carolina, United States of America; 9 Daniel B. Warnell School of Forestry and Natural Resources, University of Georgia, Athens, Georgia, United States of America; Florida State University, UNITED STATES

## Abstract

Population assessments conducted at reproductive sites of migratory species necessitate understanding the foraging-area origins of breeding individuals. Without this information, efforts to contextualize changes in breeding populations and develop effective management strategies are compromised. We used stable isotope analysis of tissue samples collected from loggerhead sea turtles (*Caretta caretta*) nesting at seven sites in the Northern Recovery Unit (NRU) of the eastern United States (North Carolina, South Carolina and Georgia) to assign females to three separate foraging areas in the Northwest Atlantic Ocean (NWA). We found that the majority of the females at NRU nesting sites (84.4%) use more northern foraging areas in the Mid-Atlantic Bight, while fewer females use more proximate foraging areas in the South Atlantic Bight (13.4%) and more southerly foraging areas in the Subtropical Northwest Atlantic (2.2%). We did not find significant latitudinal or temporal trends in the proportions of NRU females originating from different foraging areas. Combining these findings with previous data from stable isotope and satellite tracking studies across NWA nesting sites showed that variation in the proportion of adult loggerheads originating from different foraging areas is primarily related differences between recovery units: individuals in the NRU primarily use the Mid-Atlantic Bight foraging area, while individuals from the three Florida recovery units primarily use the Subtropical Northwest Atlantic and Eastern Gulf of Mexico foraging areas. Because each foraging area is associated with its own distinct ecological characteristics, environmental fluctuations and anthropogenic threats that affect the abundance and productivity of individuals at nesting sites, this information is critical for accurately evaluating population trends and developing effective region-specific management strategies.

## Introduction

Reproductive sites where migratory species congregate offer valuable opportunities to conduct population assessments of threatened species (e.g., fish spawning sites, bird rookeries, whale and ungulate calving grounds, and marine turtle nesting beaches) [1]. Foraging grounds used during non-reproductive periods are often expansive, remote, and inaccessible, which prevent direct monitoring of individuals during most of their lives. For this reason, studies at breeding sites can provide important information for estimating demographic rates, assessing population trends and evaluating population-level responses to management strategies [2]. However, breeding aggregations are frequently composed of individuals that use different foraging areas, each associated with a different migratory distance and route, and distinguished by its own ecological characteristics, environmental conditions, anthropogenic threats, and management actions. Understanding the foraging-area composition of breeding aggregations is therefore critical for contextualizing changes in population abundance, productivity, and stability over time.

**Fig 1:**
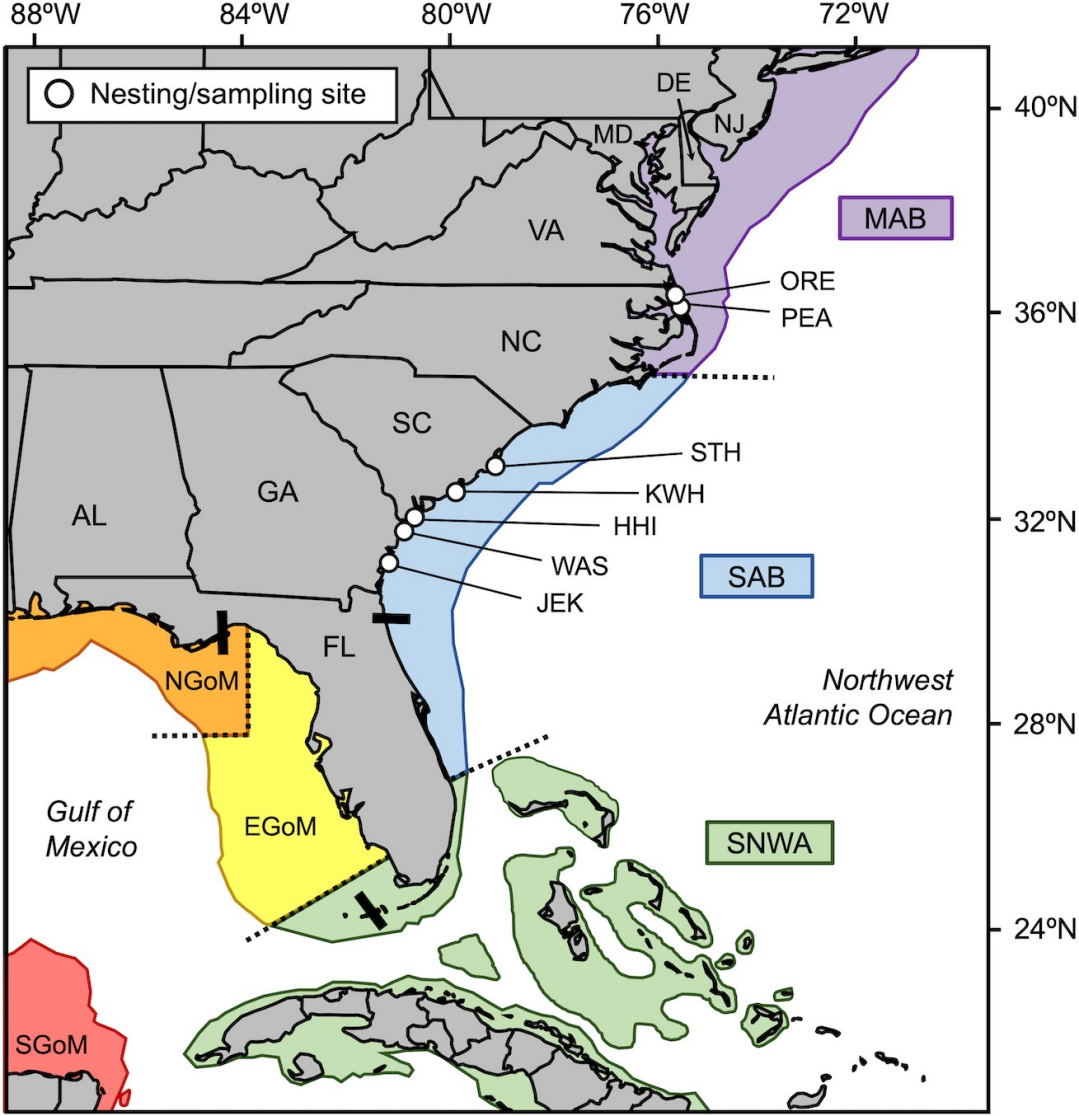
Map of nesting/sampling sites and foraging areas. Map showing the locations of the seven nesting/sampling sites (*open circles*) and six foraging areas used by loggerhead turtles in the Northwest Atlantic Ocean (*colored areas* separated by *dashed lines*). *Black bars* separate beaches used by different recovery units: Northern, Peninsular Florida, Dry Tortugas, Northern Gulf. Nesting/sampling sites: *ORE*, north of Oregon Inlet, North Carolina (NC); *PEA*, Pea Island, NC; *STH*, South Island, South Carolina (SC); *KWH*; Kiawah Island, SC; *HHI*, Hilton Head Island, SC; *WAS*, Wassaw Island, Georgia (GA); *JEK*, Jekyll Island, GA. Foraging areas: *MAB*, Mid-Atlantic Bight; *SAB*, South Atlantic Bight; *SNWA*, Subtropical Northwest Atlantic; *EGoM*, Eastern Gulf of Mexico; *NGoM*, Northern Gulf of Mexico; *SGoM*, Southern Gulf of Mexico. Base map generated using the SEATURTLE.ORG Maptool [17].

Loggerhead sea turtles (*Caretta caretta*) nesting in the Northwest Atlantic Ocean (NWA) are separated into five recovery units based on phylogeographic isolation and geopolitical boundaries [3–5]: (1) Northern Recovery Unit (NRU) nest on beaches from Maryland to North Florida, (2) Peninsular Florida Recovery Unit (PFRU) nest on beaches from central eastern Florida to central western Florida, (3) Dry Tortugas Recovery Unit (DTRU) nest on islands west of the Florida Keys, (4) Northern Gulf of Mexico Recovery Unit (NGMRU) nest in beaches from the Florida Panhandle through Texas, and (5) Greater Caribbean Recovery Unit (GCRU) nest in Mexico and throughout the rest of Greater Caribbean. Turtles from these recovery units display differential use of six broad foraging areas ranging from the waters off Nova Scotia in the north to the Yucatan Peninsula in the south [6–9] (Fig 1). Because individuals show site fidelity to foraging areas [6,10–12] and environmental factors such as resource availability, temperature, and ocean productivity vary among the key biogeographic regions used by loggerheads [13–14], variation in abundance and productivity at different nesting beaches may be attributed to shifts in the composition of adult females originating from different foraging areas [15–16]. Additionally, mortality threats vary among foraging areas, which could lead to regional differences in survival and recruitment in the breeding population. Determining the foraging-area origins of female loggerheads at different nesting sites across the NWA is therefore critical for evaluating trends in breeding population abundance and developing region-specific management strategies.

Data from satellite telemetry and isotopic analysis of epidermal tissue show that NRU turtles tend to use three foraging areas in the NWA: (1) Mid-Atlantic Bight (MAB), (2) South Atlantic Bight (SAB), and (3) Subtropical Northwest Atlantic (SNWA) [14–15,18]. The MAB extends from Cape Cod in Massachusetts to Cape Hatteras in North Carolina (NC); the SAB extends from Cape Hatteras, NC to West Palm Beach in Florida (FL) and includes the majority of the nesting area used by NRU loggerheads [3]; the SNWA extends from West Palm Beach, FL to Naples, FL and includes the Florida Keys and The Bahamas (Fig 1). The distinct biotic and abiotic features of each biogeographic region [13,19] contributes to distinct isotopic values in lower trophic levels, which are then transferred up the food web and incorporated into the tissues of turtles using each area [20]. Because isotopic turnover is relatively slow in sea turtles (at least 4 months in epidermal tissue) [21], skin samples collected at nesting sites reflect the diet of turtles at foraging areas prior to their breeding migration [14–15,18,22–23]. Similar to skin samples, the isotopic composition of egg yolks collected at nesting beaches can be used to assign nesting females to distinct foraging areas [24–28].

Previous studies on NRU loggerheads show that the majority of turtles (70–80%) nesting on Bald Head Island, NC and Wassaw Island in Georgia (GA) use the MAB foraging area, while fewer use the SAB foraging area (15–25%) and even fewer use the SNWA foraging area (~5%) [14–15,29]. The first goal of this study was to evaluate the consistency of this pattern across the nesting area used by NRU loggerheads, including nesting sites located within the MAB foraging area (north of Cape Hatteras, NC). The second goal of this study was to combine these new data with previous data from stable isotope and satellite tracking studies across NRU and NWA nesting and breeding sites to gain a deeper understanding of the spatial patterns in habitat use and migration of this important and threatened population.

## Methods

### Ethics statement

The animal use protocol for this research was reviewed and approved by the University of Florida Institutional Animal Care and Use Committee (IACUC protocol #20081985 and #201101985). Procedures were permitted by the individual state sea turtle management agencies under the authority of the United States Fish and Wildlife Service (North Carolina Wildlife Resources Commission, South Carolina Department of Natural Resources, and Georgia Department of Natural Resources).

### Study sites and sample collection

Egg yolk samples were collected from 596 clutches laid by loggerhead turtles during the 2012 and 2013 nesting seasons (May-August) at six nesting sites (Table 1A; Fig 1): north of Oregon Inlet (ORE; 35.77–36.54° N, 75.54–75.86° W; only 2012), and Pea Island (PEA; 35.71° N, 75.49° W) in North Carolina (NC), South Island (STH; 33.19° N, 79.19° W), Kiawah Island (KWH; 32.61° N, 80.08° W), and Hilton Head Island (HHI; 32.18° N, 80.74° W) in South Carolina (SC), and Wassaw Island (WAS; 31.84° N, 80.98° W) in Georgia (GA). Additionally, 150 skin samples were collected from adult female loggerhead turtles during the 2011–2013 nesting seasons at Jekyll Island, GA (JEK; 31.07° N, 81.42° W) (Table 1A; Fig 1). Egg yolk samples were collected within 10-12h of clutch deposition and placed in 95% ethanol, while skin samples (6mm biopsy punches) were collected from the shoulder region of each turtle following oviposition or failed nesting and placed in 70% ethanol. All samples were stored at room temperature prior to sample preparation and analysis in January 2014 (6–19 months after collection).

**Table 1:** Summary of foraging-area assignments based on stable isotope analysis for loggerheads nesting in the NWA.

Dataset	Site code	Latitude (°N)	Year	Total samples	Not assigned (<0.80)	Assigned duplicate (≥0.80)^a^	Assigned (≥0.80) to one of three foraging areas (proportion)		
Nesting site							MAB	SAB	SNWA
**(A) New data**									
N. of Oregon Inlet, NC	ORE	36.27	2012	18	0	1	12 (0.71)	4 (0.24)	1 (0.06)
Pea Island, NC	PEA	35.71	2012	18	1	1	11 (0.69)	4 (0.25)	1 (0.06)
	PEA	35.71	2013	6	0	0	4 (0.67)	2 (0.33)	0 (0.00)
South Island, SC	STH	33.19	2012	42	7	0	30 (0.86)	5 (0.14)	0 (0.00)
	STH	33.19	2013	51	12	0	36 (0.92)	2 (0.05)	1 (0.03)
Kiawah Island, SC	KWH	32.61	2012	62	11	1	47 (0.94)	1 (0.02)	2 (0.04)
	KWH	32.61	2013	84	17	0	59 (0.88)	8 (0.12)	0 (0.00)
Hilton Head Island, SC	HHI	32.18	2012	73	11	0	52 (0.84)	9 (0.15)	1 (0.02)
	HHI	32.18	2013	94	19	1	61 (0.82)	10 (0.14)	3 (0.04)
Wassaw Island, GA	WAS	31.84	2012	55	12	2	36 (0.88)	5 (0.12)	0 (0.00)
	WAS	31.84	2013	93	13	5	71 (0.95)	2 (0.03)	2 (0.03)
Jekyll Island, GA	JEK	31.07	2011	37	4	0	25 (0.76)	7 (0.21)	1 (0.03)
	JEK	31.07	2012	62	17	0	30 (0.67)	15 (0.33)	0 (0.00)
	JEK	31.07	2013	51	9	4	31 (0.82)	6 (0.16)	1 (0.03)
TOTAL (A)				746	133 (0.18)	15	505 (0.85)	80 (0.13)	13 (0.02)
**(B) Published data from NRU (included in some analyses and Figs 2–4)**									
Bald Head Island, NC	BHI	33.86	2004	6	2	-	1 (0.25)	2 (0.50)	1 (0.25)
	BHI	33.86	2005	12	2	-	9 (0.90)	1 (0.10)	0 (0.00)
Wassaw Island, GA	WAS	31.84	2004	17	3	-	7 (0.50)	4 (0.29)	3 (0.21)
	WAS	31.84	2005	47	8	-	30 (0.77)	7 (0.18)	2 (0.05)
	WAS	31.84	2006	19	1	-	16 (0.89)	1 (0.06)	1 (0.06)
	WAS	31.84	2009	49	3	-	41 (0.89)	5 (0.11)	0 (0.00)
	WAS	31.84	2011	80	14	-	50 (0.76)	14 (0.21)	2 (0.03)
	WAS	31.84	2014	43	13	-	22 (0.73)	5 (0.17)	3 (0.10)
TOTAL (B)				273	46 (0.17)	-	176 (0.78)	39 (0.17)	12 (0.05)
NRU TOTAL (A + B)				1019	179 (0.18)		681 (0.83)	119 (0.14)	25 (0.03)
**(C) Published data from PFRU (not included in analyses, but in Figs 2 and 4)**									
Canaveral National Seashore, FL	CNS	28.79	2003	44	3	-	4 (0.10)	24 (0.58)	13 (0.32)
	CNS	28.79	2004	31	3	-	6 (0.21)	10 (0.36)	12 (0.43)
Melbourne Beach, FL	MEL	28.01	2003	60	5	-	11 (0.20)	19 (0.35)	25 (0.45)
	MEL	28.01	2004	46	1	-	2 (0.04)	18 (0.40)	25 (0.56)
Juno Beach, FL	JUN	26.88	2003	41	3	-	8 (0.21)	10 (0.26)	20 (0.53)
	JUN	26.88	2004	41	4	-	1 (0.03)	6 (0.16)	30 (0.81)
Broward County, FL	BRO	26.19	2003	47	4	-	1 (0.02)	14 (0.33)	28 (0.65)
PFRU TOTAL (C)				310	23 (0.07)	-	33 (0.12)	101 (0.35)	153 (0.53)

(A) New foraging-area assignments for 596 yolk samples (ORE, PEA, STH, KWH, HHI and WAS) and 150 skin samples (JEK) collected from loggerhead turtles nesting at seven sites in the Northern Recovery Unit (NRU; listed north to south).

(B) Previously published foraging-area assignments and proportions for 273 skin samples collected from NRU loggerheads nesting at one additional site (BHI) [14] and six additional years from WAS [15,29].

(C) Previously published foraging-area assignments and proportions for 310 skin samples collected from loggerhead turtles nesting at four sites in the Peninsular Florida Recovery Unit (PFRU; listed north to south) [14].

Abbreviations: MAB, Mid-Atlantic Bight; SAB, South-Atlantic Bight; SNWA, Subtropical Northwest Atlantic (Fig 1)

^a^ Samples collected from the same individual, either at different sites within the same season or in different seasons, *and* assigned to one of the three foraging areas with posterior probabilities ≥0.80 were excluded from analyses. All duplicate samples from the same individual were assigned to the same foraging area.

At the two southernmost sampling sites, Wassaw and Jekyll Island, GA (Fig 1), individuals encountered during nocturnal patrols were identified using either physical tags (two Inconel metal tags and one PIT tag) or unique multilocus genetic tags determined from DNA extracted from skin samples collected during each turtle’s first encounter in the study [30–31]. At these two sites, tissue samples–yolk from WAS or epidermis from JEK–were collected from each female one time per season (i.e., samples were not collected during within-season recaptures of previously sampled females identified by their tags). At the five sites in SC and NC where nocturnal tagging patrols are not conducted (ORE, PEA, STH, KWH and HHI), yolk samples were identified to individual females using the aforementioned multilocus genetic tags extracted from maternal DNA found in the shell of freshly laid eggs [32]. See Shamblin et al. [32–33] for details on genetic tagging across this subpopulation. All samples were included in subsequent stable isotope analysis (SIA) to crosscheck foraging-ground assignments when individual females were sampled at more than one site within the same season or within more than one season, but each individual was included only once when comparing the contribution of different foraging grounds to each nesting site.

### Sample preparation and analysis

Ethanol-preserved yolk samples were mixed into a homogeneous solution, then portions of each sample were pipetted into weigh boats and dried at 60°C for 24-48h. Yolk samples were not lipid-extracted prior to SIA. Instead, previous work demonstrates that untreated yolk preserved in ethanol can be converted to maternal epidermis values using the following equations: δ^13^C_skin_ = 0.58 x δ^13^C_yolk_—4.27 and δ^15^N_skin_ = 1.16 x δ^15^N_yolk_ -1.90 [27]. These equations account for the lipid normalization step, as there are separate equations for yolk that was frozen or lipid-extracted prior to SIA [27].

Skin samples were rinsed with distilled water and cleaned with isopropyl alcohol swabs, then the epidermis was separated from the dermis, homogenized using a scalpel blade and dried at 60°C for 24-48h. Skin samples were not lipid extracted prior to SIA because lipid extraction does not affect the stable isotope values of loggerhead epidermis [15].

For SIA, 0.5–0.6 mg of each sample was analyzed for δ^13^C and δ^15^N composition by combustion in an ECS 4010 elemental analyzer (Costech) interfaced via a ConFlo III to a DeltaPlus XL isotope ratio mass spectrometer (ThermoFisher Scientific) at the University of Florida Light Stable Isotope Mass Spectroscopy Laboratory. Delta notation is used to express stable isotope abundances, as defined as parts per thousand (‰) relative to the standard: δX = [(R_sample_/R_standard_)– 1] × 1000, where R_sample_ and R_standard_ are the corresponding ratios of rare to common isotopes (^13^C/^12^C and ^15^N/^14^N) in the sample and international standard, respectively. Vienna Pee Dee Belemnite was used as the standard for ^13^C, and atmospheric N_2_ for ^15^N. Working standard, L-glutamic acid USGS40 (δ^13^C = −26.39‰ and δ^15^N = −4.52‰), was used to normalize results. In addition, a reference laboratory standard, homogenized loggerhead scute (δ^13^C = −18.36‰ and δ^15^N = 7.68‰), was used to examine consistency in isotopic values in a sample similar to the samples used in this study. The analytical precision of measurements–calculated as the standard deviation of replicates from laboratory standards–was 0.14‰ for δ^13^C and 0.24‰ for δ^15^N of L-glutamic acid USGS40 (N = 77) and 0.20‰ for δ^13^C and 0.26‰ for δ^15^N of loggerhead scute (N = 29).

### Foraging area determination

Quadratic discriminant function analysis of stable isotope values was used to assign females to one of the three foraging areas known for NRU loggerheads: (1) MAB, (2) SAB, or (3) SNWA (Fig 1). The training data for the quadratic discriminant function analysis consisted of skin stable isotope values of 60 adult loggerheads from known foraging areas (either through satellite tracking or from sample collection at a foraging site) collected between 2004 and 2011 [14; S1 Table]. A uniform prior probability distribution was assumed, meaning it was equally likely the individuals could have originated from each foraging area, rather than weighting the prior probability by sample size from each area. Only females assigned to one of the three foraging areas with posterior probabilities ≥0.80 were considered. This constraint translates to an eightfold improvement in odds over random assignment [34].

Using stable isotope values to determine foraging origin of animals depends on the use of isotopic measurements at temporal and spatial scales relevant to the movement of the animals of interest [35]. Several factors may affect the isotopic values of sea turtles over time (e.g., baseline variation, prey composition, food stress, etc.), which could confound foraging area determination. The effect of these factors on the isotopic values turtle tissues are not yet well understood or have not been described. However, despite these potential difficulties, the highly structured and predictable isotopic values encountered in the turtles with known foraging areas sampled over various years allowed for foraging area determination of sea turtles in the NWA in this study.

### Statistical analyses

Pearson’s Chi-squared tests with *p*-values computed through Monte Carlo simulation (based on 2,000 replicates) were used to compare the proportion of turtles originating from different foraging areas. First, we compared the inter-annual variation within each applicable nesting site in this study: 2012–2013 for PEA, STH, KWH, HHI and WAS, and 2011–2013 for JEK (Bonferroni-corrected alpha = 0.008 for six comparisons; ORE sampled only in 2012). Second, we pooled all years within each site and tested for differences between sites by making all pairwise comparisons between the seven sites in this study, Bald Head Island (BHI), NC (2004–2005), and an eighth “site” called WAS* that included additional data for WAS collected between 2004–2014 (Table 1B) (Bonferroni-corrected alpha = 0.0014 for 34 comparisons). Data for BHI and WAS published in Pajuelo et al. [14], Vander Zanden et al. [15], and Price et al. [29] provide direct comparisons because these studies use the same training data and statistical constraints to assign females to different foraging areas. From these data, we also tested for trends between the latitude of each nesting site and the proportion of turtles originating from each foraging area using simple linear regressions–one analysis for each of the three foraging areas. Last, we compared the inter-annual variation across years on WAS and tested for differences between years by making all pairwise comparisons between sampled years from 2004–2014 (Bonferroni-corrected alpha = 0.002 for 28 comparisons). From these data, we also estimated trends in the proportion of turtles originating from each foraging area over time using generalized least squares with first order autocorrelated error structure [36]–one analysis for each of the three foraging areas.

### Review of stable isotope and satellite tracking studies

We conducted a two-tiered literature search to collect published articles that use either stable isotopes or satellite tracking to identify the foraging-area origins/destinations of nesting (females) or breeding (males) loggerhead turtles in the NWA. First, a structured search was conducted in Google Scholar and Web of Science using the following search terms: stable isotope, satellite, loggerhead, *Caretta* and Atlantic. Then, an unstructured search was conducted by reviewing the reference lists of all relevant publications from the structured search. Theses and dissertations were included, but conference presentations and reports were not. We reviewed all potential references and retained only those involving loggerheads nesting or breeding in the NWA that were either assigned to foraging areas using stable isotope analysis or satellite tracked to foraging areas during post-breeding migrations.

For each nesting or breeding site, we collated the number of individuals that were assigned or tracked to each foraging area (Fig 1), keeping stable isotope and satellite tracking studies separate. We combined the new dataset in this study with other studies that use stable isotopes. For satellite-tracking studies, we counted each satellite-tracked individual only once (to the best of our knowledge). When satellite tracking data were included in multiple publications, we used the foraging-area destinations from the most recent study because they frequently involve different and/or more robust statistical analyses, as well as additional unpublished tracks collected in subsequent years.

## Results

### New foraging-area assignments in the NRU

Of the 596 yolk samples (ORE, PEA, STH, KWH, HHI and WAS) and 150 skin samples (JEK) collected from individual females and analyzed for stable isotopes, 493 yolk samples (83%) and 120 skin samples (80%) were assigned to one of the three foraging areas with posterior probabilities ≥0.80 (Table 1A; S1 Fig). A total of 133 samples (18%) could not be assigned to a foraging area–posterior probability <0.80 for all foraging areas. Twenty-five individuals were sampled twice either at more than one site within the same season or within more than one season: 15 individuals had both samples assigned to a foraging area (posterior probability ≥0.80 for one area), six had only one sample assigned, and four had neither sample assigned (posterior probability <0.80 for all areas). All 15 individuals with assignable duplicate samples were assigned to the same foraging area in both samples (14 MAB and 1 SNWA). After removing unassignable duplicates and assignable duplicates collected during a second sampling occasion, 482 yolk samples (81%) and 116 skin samples (77%) were attributed to 598 individuals, each assigned to one of the three foraging areas with posterior probabilities ≥0.80 and included only once in the dataset. Stable isotope ratios (following appropriate egg yolk to skin conversions) and posterior probabilities for foraging-area assignment of each sample are provided in S2 Table (metadata for S2 Table provided in S1 File).

Within each nesting site sampled in this study, there were no annual differences in the proportion of females originating from different foraging areas (Pearson’s chi-square tests: ORE, only 2012; PEA, *χ*^2^ = 0.013, df = 2, *P* ~ 1.0; STH, *χ*^2^ = 2.62, df = 2, *P* = 0.22; KWH, *χ*^2^ = 6.47, df = 2, *P* = 0.02; HHI, *χ*^2^ = 0.72, df = 2, *P* = 0.76; WAS, *χ*^2^ = 5.2, df = 2, *P* = 0.07; JEK, *χ*^2^ = 4.95, df = 4, *P* = 0.32; Bonferroni-corrected alpha = 0.008 for six comparisons). Therefore, data collected in different years were pooled for spatial analyses (2012–2013 for ORE, PEA, STH, KWH, HHI and WAS, and 2011–2013 for JEK).

**Fig 2:**
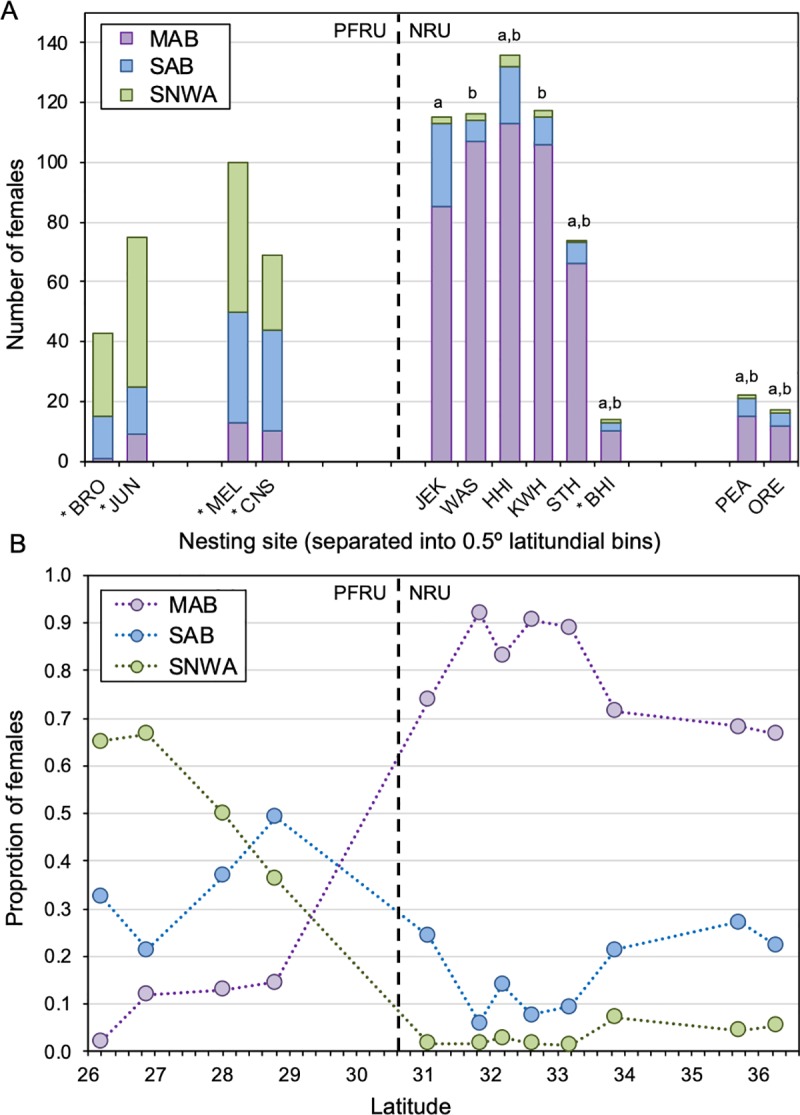
Latitudinal variation in foraging-area origin among nesting sites. (A) Bar chart and (B) line graph displaying latitudinal variation among nesting sites in the proportion of female loggerheads originating from three different foraging areas: *MAB*, Mid-Atlantic Bight; *SAB*, South Atlantic Bight; *SNWA*, Subtropical Northwest Atlantic. (A) *Letters* indicate statistical differences among eight nesting sites in the Northern Recovery Unit (NRU): *ORE*, north of Oregon Inlet; *PEA*, Pea Island; *BHI*, Bald Head Island; *STH*, South Island; *KWH*; Kiawah Island; *HHI*, Hilton Head Island; *WAS*, Wassaw Island; *JEK*, Jekyll Island. *Asterisk* indicates previously published foraging-area assignments for females nesting at *BHI* and four nesting sites in the Peninsular Florida Recovery Unit (PFRU: *CNS*, Canaveral National Seashore; *MEL*, Melbourne Beach; *JUN*, Juno Beach; *BRO*, Broward County beaches) that were included because foraging areas were assigned using the same training data and statistical constraints [14]. (B) There were no significant trends between the latitude of NRU nesting sites and the proportion of females originating from each of the three different foraging areas (simple linear regression; one analysis for each of the three foraging areas).

Overall, the majority of the assigned females nesting at the seven study sites was assigned to the northern MAB foraging area (505 out of 598 females; 84.4%) (Table 1). Eighty females (13.4%) were assigned to the central SAB foraging area, and 13 females (2.2%) were assigned to the southern SNWA foraging area. These proportions were qualitatively similar to previous studies conducted on NRU loggerheads in different years, but very different than loggerheads nesting in Peninsular Florida (Table 1; Fig 2). Florida data from Pajuelo et al. [14] are shown in Table 1C and Fig 2 because the same training data and statistical constraint (posterior probability ≥0.80) were used to assign females to different foraging areas. Ceriani et al. [16,22] used different training data and a less stringent statistical constraint (posterior probability ≥0.60) for foraging-area assignments and are therefore not included in Table 1 or Fig 2 but are included in the stable isotope summary below.

Among NRU nesting sites, the proportion of females using different foraging areas was significantly different between (1) JEK and KWH and (2) JEK and WAS (dark gray cells, Table 2; Fig 2A). While the relative proportions remained the same, these differences are primarily attributable to a slightly higher proportion of females nesting on JEK (the southernmost sampling site) that use the central SAB foraging area versus the more northerly MAB foraging area. Despite some significant differences between sites, as well as smaller samples sizes at the two northern sites (ORE and PEA), there were no significant trends between the latitude of the nesting site and the proportion of females originating from each of the three different foraging areas (simple linear regression; MAB: *y* = -0.034*x* + 1.94, *F*_6_ = 3.32, *P* = 0.12; SAB: *y* = 0.026*x* – 0.69, *F*_6_ = 2.44, *P* = 0.17; SNWA: *y* = 0.009*x* – 0.27, *F*_6_ = 6.30, *P* = 0.05; Fig 2B).

**Table 2:** Pairwise comparisons of foraging-area proportions among NRU nesting sites (listed north to south).

Site		ORE	PEA	BHI	STH	KWH	HHI	WAS	WAS*	JEK
			Monte Carlo simulated *p*-value (2000 replicates)							
**ORE**			0.95	1	0.09	0.07	0.46	0.06	0.49	0.58
**PEA**	*X*-squared statistic	0.09		1	0.07	0.02	0.29	0.004	0.14	0.67
**BHI**		0.03	0.23		0.15	0.07	0.33	0.06	0.48	0.42
**STH**		4.11	5.69	3.65		0.89	0.52	0.73	0.35	0.03
**KWH**		5.66	8.34	4.71	0.22		0.21	0.93	0.14	0.001
**HHI**		1.61	2.79	1.36	1.51	3.05		0.09	0.87	0.10
**WAS**		7.40	10.74	6.04	0.81	0.25	4.81		0.06	0.0005
WAS*		1.75	3.55	1.25	2.09	4.02	0.33	5.90		0.01
**JEK**		1.15	0.81	1.62	6.74	12.05	4.62	15.12	8.88	

Pearson’s chi-square test statistics (below diagonal) and associated *p*-values simulated following 2000 Monte Carlo replicates (above diagonal) for all pairwise comparisons (df = 2) between the proportions of females at eight nesting sites in the Northern Recovery Unit that originated from three different foraging areas. Gray cells highlight significant differences at a Bonferroni-corrected alpha = 0.0014 following 34 comparisons.

Abbreviations: ORE, North of Oregon Inlet, NC; PEA, Pea Island, NC; BHI, Bald Head Island, NC; STH, South Island, SC; KWH, Kiawah Island, SC; HHI, Hilton Head Island, SC; WAS, Wassaw Island (2012–2013), GA; WAS*, Wassaw Island (2004–2014); JEK, Jekyll Island, GA (Fig 1). See Table 1 for foraging-ground proportions by year.

**Fig 3:**
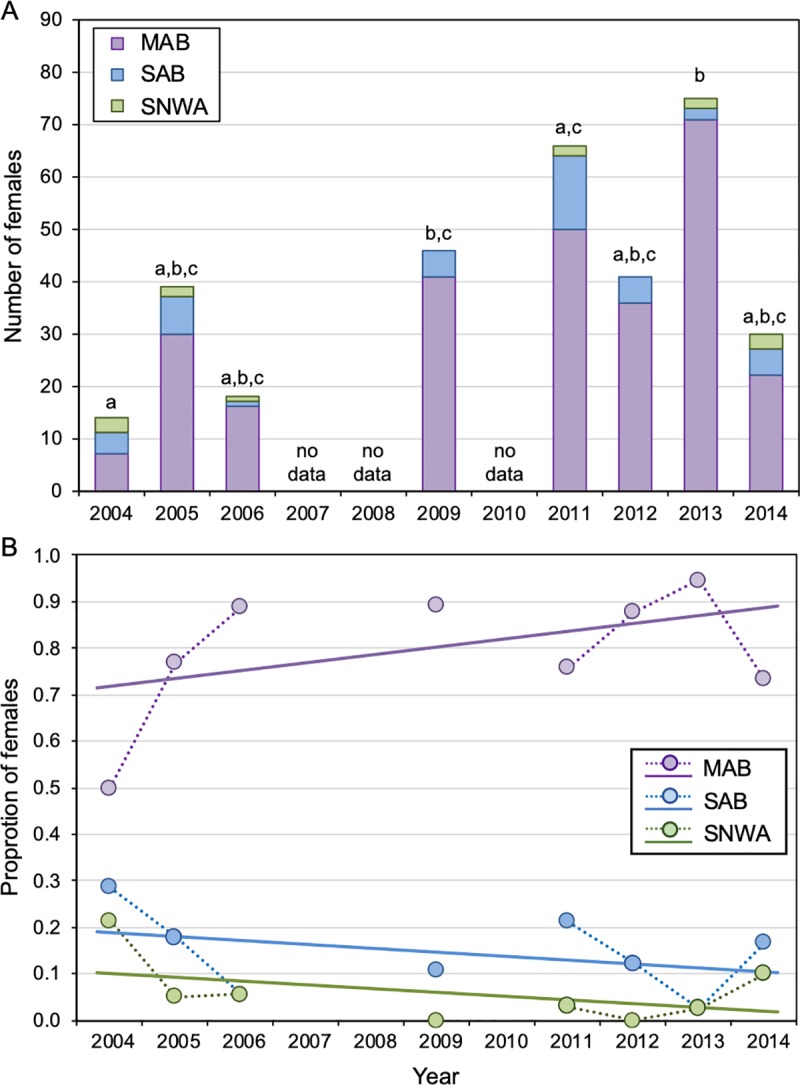
Temporal variation in foraging-area origins for Wassaw Island, GA. (A) Bar chart and (B) line graph displaying temporal variation in the proportion of female loggerheads nesting on Wassaw Island, GA originating from three different foraging areas: *MAB*, Mid-Atlantic Bight; *SAB*, South Atlantic Bight; *SNWA*, Subtropical Northwest Atlantic. (A) *Letters* indicate statistical differences among sampled years (Pearson’s Chi-squared tests; all pairwise comparisons). (B) There were no significant trends over time in the proportion of females originating from the three foraging areas (generalized least squares with first order autocorrelated error structure; one analysis for each of the three foraging areas). Previously published data were included for 2004–2011 from Vander Zanden et al. [15] and 2014 from Price et al. [29].

Among the eight sampled nesting seasons between 2004–2014 on WAS, there was a significant difference in the proportion of females using different foraging areas (Pearson’s chi-square tests; *χ*^2^ = 36.25, df = 14, *P* = 0.0035). The proportion of females using different foraging areas was significantly different between (1) 2004 and 2009, (2) 2004 and 2013, (3) 2011 and 2013 (dark gray cells, Table 3; Fig 3A). Despite some differences among years, as well as small samples sizes in two of the early years (2004 and 2006), there were no significant trends over time in the proportion of WAS females originating from the three foraging areas (generalized least squares: MAB, t_8_ = 1.39, *p*-value = 0.22; SAB, t_8_ = -1.16, *p*-value = 0.29; SNWA, t_8_ = -1.33, *p*-value = 0.23; Fig 3B).

**Table 3:** Pairwise comparisons of foraging-area proportions among years for Wassaw Island, GA.

Year		2004	2005	2006	2009	2011	2012	2013	2014
			Monte Carlo simulated *p*-value (2000 replicates)						
**2004**			0.12	0.06	0.002	0.03	0.003	0.0005	0.35
**2005**	*X*-squared statistic	4.53		0.55	0.16	0.86	0.28	0.007	0.83
**2006**		5.91	1.57		0.31	0.25	0.32	1	0.47
**2009**		14.15	3.48	2.95		0.15	1	0.11	0.06
**2011**		7.61	0.42	2.50	3.70		0.32	0.0005	0.42
**2012**		12.4	2.83	2.82	0	2.86		0.07	0.10
**2013**		21.82	8.94	0.80	4.64	12.12	5.22		0.004
**2014**		2.36	0.60	1.72	5.61	2.16	4.79	9.82	

Pearson’s chi-square test statistics (below diagonal) and associated *p*-values simulated following 2000 Monte Carlo replicates (above diagonal) for all pairwise comparisons (df = 2) between the proportions of females sampled each year on Wassaw Island, GA that originated from three different foraging area. Gray cells highlight significant differences at a Bonferroni-corrected alpha = 0.002 following 28 comparisons. See Table 1 for foraging-ground proportions by site and by year.

### Patterns within the NRU and across the NWA

**Fig 4:**
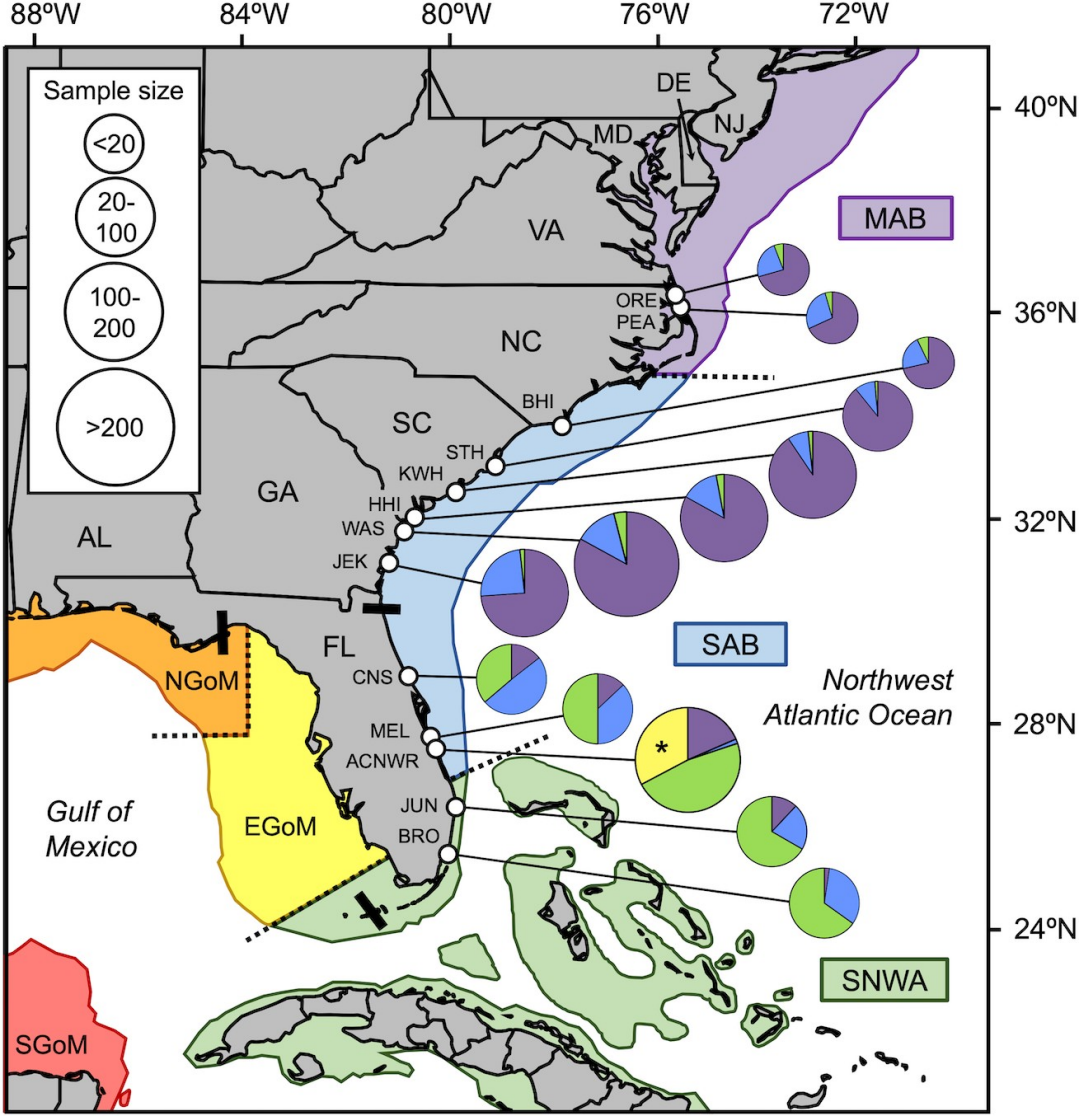
Summary of foraging-area origins based on stable isotopes from female loggerheads nesting in the NWA. *Black bars* separate beaches used by different recovery units: Northern, Peninsular Florida, Dry Tortugas, Northern Gulf. Nesting/sampling sites: *ORE*, north of Oregon Inlet, North Carolina (NC); *PEA*, Pea Island, NC; *BHI*, Bald Head Island, NC; *STH*, South Island, South Carolina (SC); *KWH*; Kiawah Island, SC; *HHI*, Hilton Head Island, SC; *WAS*, Wassaw Island, Georgia (GA); *JEK*, Jekyll Island, GA; *CNS*, Canaveral National Seashore, Florida (FL); *MEL*, Melbourne Beach, FL; *ACNWR*, Archie Carr National Wildlife Refuge, FL (*Asterisk* indicates stable isotope data that were not included in Fig 2 due to differing statistical approaches); *JUN*, Juno Beach, FL; *BRO*, Broward County beaches, FL. Foraging areas (*colored areas* separated by *dashed lines*): *MAB*, Mid-Atlantic Bight; *SAB*, South Atlantic Bight; *SNWA*, Subtropical Northwest Atlantic; *EGoM*, Eastern Gulf of Mexico; *NGoM*, Northern Gulf of Mexico; *SGoM*, Southern Gulf of Mexico. Data are from Ceriani et al. [16,22], Pajuelo et al. [14], Price et al. [29], Vander Zanden et al. [15], and this study (S3 Table). Base map generated using the SEATURTLE.ORG Maptool [17].

**Fig 5:**
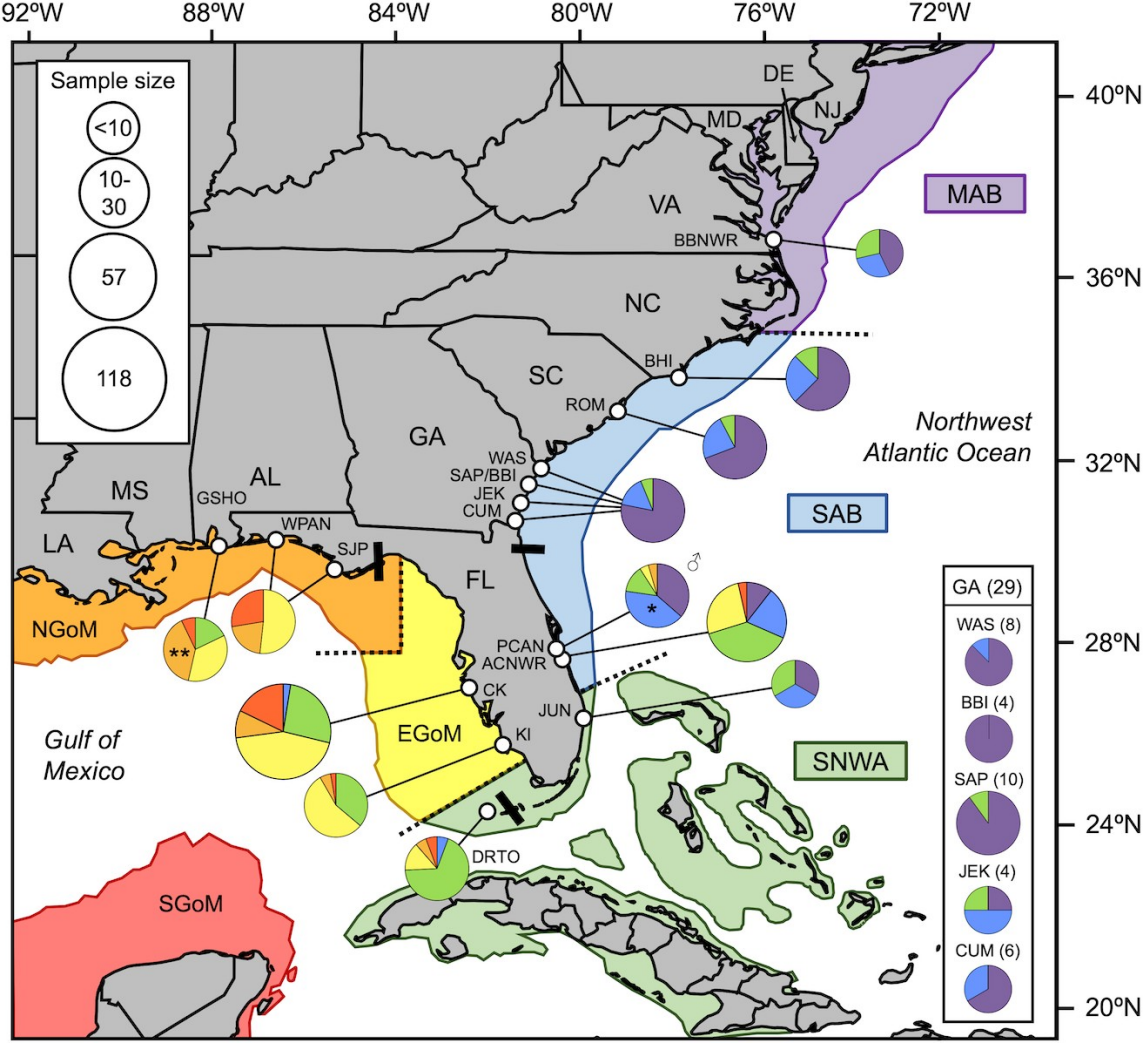
Summary of foraging-area destinations of adult loggerheads satellite tracked during post-breeding migrations in the NWA. *Black bars* separate beaches used by different recovery units: Northern, Peninsular Florida, Dry Tortugas, Northern Gulf. *B*reeding sites: *BBNWR*, Back Bay National Wildlife Refuge, Virginia (VA); *BHI*, Bald Head Island, North Carolina (NC); *ROM*, Cape Romain, South Carolina (SC); *WAS*, Wassaw Island, Georgia (GA); *SAP*, Sapelo Island, GA; *BBI*, Blackbeard Island, GA; *JEK*, Jekyll Island, GA; *CUM*, Cumberland Island, GA; *PCAN*, Port Canaveral Shipping Channel, Florida (FL; *Asterisk* indicates reproductively active adult males only; four non-reproductively active males excluded); *ACNWR*, Archie Carr National Wildlife Refuge, FL; *JUN*, Juno Beach, FL; *DRTO*, Dry Tortugas, FL; *KI*, Keewaydin Island, FL: *CK*, Casey Key, FL; *SJP*, St. Joseph Peninsula, FL; *WPAN*, Western Panhandle, FL; *GSHO*, Gulf Shores, Alabama (AL; *Double asterisk* includes one female that migrated in the Western Gulf of Mexico). Foraging areas: *MAB*, Mid-Atlantic Bight; *SAB*, South Atlantic Bight; *SNWA*, Subtropical Northwest Atlantic; *EGoM*, Eastern Gulf of Mexico; *NGoM*, Northern Gulf of Mexico; *SGoM*, Southern Gulf of Mexico. Data are from Arendt et al. [37–38], Ceriani et al. [9,16,22], Dodd and Byles [39], Evans et al. [40], Foley et al. [7], Girard et al. [41], Griffin et al. [42], Hardy et al. [43], Hart et al. [12,44–45], Hawkes et al. [6], Lamont et al. [46], Mansfield [47], Phillips [48], Plotkin and Spotila [49], Tucker et al. [8], Vander Zanden et al. [50] (S3 Table). Base map generated using the SEATURTLE.ORG Maptool [17].

Among 24 published articles identified in our literature search (S3 Table), we found 1,398 loggerheads from 13 nesting sites that were assigned to an NWA foraging area using stable isotope analysis (Fig 4) and 408 loggerheads from 16 nesting/breeding sites that were tracked to an NWA foraging area using satellite telemetry (Fig 5). Spatial patterns in the proportion of individuals that use different foraging areas were mostly congruent between stable isotope and satellite tracking studies. Stable isotopes have not been used to infer foraging-area origins for female loggerheads nesting in Dry Tortugas or Gulf of Mexico (Fig 4), making satellite tracking data the only means to compare patterns across all recovery units in the US. Foraging-area proportions from nesting sites with small samples sizes (smaller pie charts on Figs 4 and 5) are less robust because the foraging-area origin of each individual carries proportionately more weight for describing the overall pattern. Because satellite-tracking studies often suffer from small sample sizes, these patterns should be interpreted with more caution.

Among nesting sites in GA, SC, NC, and VA (NRU), the majority of females use the MAB foraging area, while fewer use the SAB and SNWA (stable isotopes: 83% MAB > 14% SAB > 3% SNWA; satellite tracking: 68% MAB > 21% SAB > 11% SNWA) (Figs 4 and 5). Among satellite tracking studies, the percentage of NRU females tracked to the MAB vs. SAB and SNWA decreased from south to north on a state-by-state basis: GA (76% MAB, 24% SAB and SNWA) > SC (69%, 31%) > NC (63%, 37%) > VA (43%, 57%) (simple linear regression; MAB: *y* = -0.07*x* + 2.82, *F*_3_ = 204.7, *P* = 0.005) (Fig 5).

Among nesting sites in eastern FL (PFRU), the majority of females use the SNWA foraging area, while fewer use the MAB, SAB, and EGoM (stable isotopes: 50% SNWA > 18% SAB > 16% EGoM > 15% MAB; satellite tracking: 38% SNWA > 23% EGoM > 22% SAB > 13% MAB) (Figs 4 and 5). Conversely, the majority of males breeding in eastern FL use the SAB and MAB foraging areas (satellite tracking: 41% SAB > 36% MAB > 14% SNWA > 5% EGoM). Among stable isotope assignments, the proportion of eastern PFRU females assigned to the SNWA increased from north to south: CNS (36%) < MEL (50%) = ACNWR (47%) < JUN (67%) = BRO (65%) (simple linear regression; SAB: *y* = -7.47*x* + 31.5, *F*_3_ = 26.9, *P* = 0.014) (Fig 4).

Among satellite tracking studies in western FL (PFRU), the majority of females use the EGoM foraging area, while fewer use the SNWA, NGoM, and SGoM (satellite tracking: 47% EGoM > 29% SNWA > 14% NGoM > 8% SGoM) (Fig 5). The majority of females that were satellite tracked after nesting in the Dry Tortugas, FL (DTRU) use the SNWA foraging area (68%), while the remaining females use four other foraging areas excluding the MAB. The majority of females that were satellite tracked after nesting in the FL panhandle and AL (NGMRU) use the EGoM and NGoM foraging areas, while fewer use the SNWA and SGoM (satellite tracking: 44% EGoM > 30% NGoM > 18% SGoM > 9% SNWA).

## Discussion

### Patterns within the NRU

Our data highlight the importance of the MAB foraging area for the long-term productivity and stability of the NRU loggerhead subpopulation. Variation in the proportion of females assigned to different foraging areas was subtle based on isotopic data with the majority of females at all eight nesting sites originating from the northern MAB foraging area (Fig 4). The importance of the MAB foraging area to NRU loggerheads is also reflected in the post-nesting migrations of satellite-tracked females (Fig 5). Whereas we detected some differences among NRU nesting sites and among nesting seasons at one long-term NRU site (Wassaw Island, GA) in our new dataset, these differences were not associated with significant latitudinal or temporal trends in the proportions of females originating from different foraging areas (Figs 3B and 4B). However, in the satellite-tracking data there was a significant latitudinal trend in which the proportion of post-nesting females that migrate to the MAB vs. SAB and SNWA decreased from south to north. These differences might be attributable to a subtle, long-term increase in the proportion of NRU females using the MAB foraging area that has swamped any latitudinal pattern in more recent years.

Satellite tracking data that were used to determine the post-nesting foraging-area destinations in Fig 5 were collected between 1992–2008 (79% before 2005), whereas studies that used isotopic data to assign nesting females to foraging areas (Fig 4) were conducted between 2004–2014 (72% after 2012) (S3 Table). Although the trend was not statistically significant and early years may be biased by small sample sizes, the proportion of females nesting on Wassaw Island that were assigned to the MAB shows a similar increasing pattern over time (Fig 3B). Therefore, the temporal increase in the proportion of NRU females using the MAB foraging area might be a true biological shift, not an artifact of using different methodologies in different years (satellite tracking from 1992–2008 vs. stable isotopes from 2004–2014). Because this shift coincides with a substantial increase in the total number of nests laid at NRU nesting sites since the early- to mid-2000s [51–52], the health and productivity of the MAB has clearly supported the ongoing recovery of the NRU loggerhead subpopulation. Patterns among isotopic data over this 10-year period assume that there has not been a shift in the isotopic baseline, a hypothesis that has not been tested. Nevertheless, even in the absence of an increasing trend, management actions and conservation efforts aimed to maintain the viability of foraging habitats in the MAB for NRU loggerheads and minimize threats to sea turtle survival in the MAB are therefore critical for this subpopulation’s long-term stability and continued recovery [3,53–54].

Post-nesting loggerheads that use the MAB foraging area migrate north within the continental shelf through a relatively narrow corridor along the North Carolina coast before occupying localized ‘summertime’ foraging sites between North Carolina and New Jersey, including productive habitats within the Chesapeake and Delaware bays [6,42]. In the winter months (November-March), these turtles migrate south along the same corridor to overwintering sites within the northern end of the SAB, where they can enter warmer waters adjacent to the Gulf Stream while minimizing the migratory distance, time and energy required to return to their northern foraging sites when water temperatures rise in the spring [6,42,55]. Similar seasonal MAB-SAB shuttling along this “migratory bottleneck” is exhibited by immature loggerheads [54].

Stable isotope data suggest that although these females occupy habitats in the SAB for part of the year, the majority of nutrients consumed and assimilated in the time period represented by the tissue reflect habitats in the MAB [15]. The frequency of this ‘seasonal’ MAB foraging strategy in NRU loggerheads suggests that the quality of foraging habitats in the MAB offsets the negative consequences associated with seasonal migrations [42,54], as well as possible bouts of winter fasting [55]. Indeed, the MAB is one of the most productive marine regions in the world [13], and turtles nesting in Georgia that forage in the MAB are larger and have larger clutch sizes and the same average remigration interval than turtles that remain in the SAB year-round [15,29]. Therefore, despite their longer northward migrations and seasonal movements, NRU females that forage in the MAB tend to have higher fecundity and likely contribute disproportionately more offspring to future generations.

NRU loggerheads that use the SAB foraging area move relatively short distances after nesting–both to the north and south of their nesting beaches–to foraging sites distributed within the continental shelf from North Carolina to central Florida. These turtles either remain in localized foraging sites year-round or undergo seasonal shuttling between summertime foraging sites along the inner shelf (<20m) and adjacent overwintering sites in the mid- or outer shelf (>20m) [42]. Similar to the seasonal north-south movements by turtles in the MAB, seasonal west-east movements by turtles in the SAB are associated with changes in water temperature and winter movements towards the warmer waters adjacent to the Gulf Stream for thermoregulation [42]. While benthic community characteristics and productivity in these areas are poorly understood, turtles that nest *and* forage in the SAB (‘residents’) do not appear to reinvest the energy needed for longer migrations into increases in growth and reproduction. Turtles using the SAB are in fact smaller and less fecund than turtles using the MAB [15]. Persistent differences in regional productivity might help explain why the proportion of females using the SAB is lower, and possibly decreasing, relative to the MAB.

The Subtropical Northwest Atlantic (SNWA) and Eastern Gulf of Mexico (EGoM) foraging areas support the majority of adult loggerheads in the Peninsular FL Recovery Unit (PFRU), making these areas globally important to the survival of the species (see below). However, very few NRU loggerheads use these foraging areas based on data from stable isotopes (Fig 4), satellite telemetry (Fig 5), as well as tag returns [56–58]. Those NRU turtles that do use the SNWA foraging area undergo lengthy post-nesting migrations southward along the continental shelf, often through a narrow corridor between south Florida and the Gulf Stream, before taking up year-round residence in localized home ranges in either the FL Keys or The Bahamas [6,42]. The latter of these females, along with a small number that make temporary oceanic forays adjacent to the MAB and SAB, represent rare instances when adult NRU loggerheads leave US waters.

Similar to NRU females that use the MAB foraging area, NRU females that use the SNWA are larger and more fecund than females that use the SAB [15]. Despite substantial differences in migratory direction and foraging strategy, NRU females that use MAB and SNWA foraging areas do not show differences in body size or clutch size. While NRU females from the MAB tend to have shorter remigration intervals than those from the SNWA [15], these differences may be due to small sample size (too few SNWA females) or to differences in site fidelity that artificially increase remigration intervals when nesting events/seasons are missed at a focal nesting site (e.g., Wassaw Island, GA). Additional work is needed to more thoroughly evaluate not only the reproductive carry-over effects of different foraging areas used by NRU loggerheads (e.g., clutch size, clutch frequency and the length of remigration intervals), but also how the use of different foraging areas affects nest-site fidelity, recruitment and adult survival.

### Patterns across the NWA

The foraging-area origins of adult loggerheads shifts quite dramatically between NRU and PFRU nesting sites (Figs 2 and 4). While male PFRU turtles at a mating site in eastern FL predominately migrate to the MAB and SAB foraging areas after breeding [38], the proportion of PFRU female turtles that use the MAB foraging area decreases moving south along the east coast of FL as the predominant foraging area shifts from the SAB to the SNWA (Figs 4 and 5). Genetic subdivision between PFRU turtles nesting in central eastern vs. southeastern FL [3,59] is accompanied by this latitudinal shift in the predominant foraging area used by nesting females–MAB/SAB to SNWA. Nevertheless, data from both stable isotopes and satellite telemetry indicate that the MAB represents an important, although secondary, foraging area for PFRU loggerheads breeding along the east coast of Florida, a globally important nesting area for this species [60].

Like NRU females, these turtles use the same continental corridor adjacent to North Carolina during pre- and post-breeding migrations, as well as seasonal movements between summertime foraging grounds north of Cape Hatteras and overwintering sites south of Cape Hatteras [22,38]. Relative to NRU females, PFRU adults that use the MAB foraging area migrate greater distances to reach their foraging grounds. Conversely, PFRU females that use the SNWA foraging area migrate shorter distances than NRU females using the same foraging area (SNWA). While these differences in migratory distance are not associated with apparent differences in body size, clutch size, or clutch success (hatching or emergence), there is evidence for a tradeoff associated with remigration interval. Females that migrate to more distant foraging areas–PFRU females using the MAB and NRU females using the SNWA–exhibit longer remigration intervals (~5yr between nesting seasons) than females that migrate to more proximate foraging areas (~3yr between nesting seasons)–PFRU females using the SNWA and NRU females using the MAB [15–16]. Assuming that differences in nest-site fidelity do not drive apparent differences in remigration interval, this tradeoff between migratory distance and remigration interval might play an important role in regulating the frequency of nesting turtles that use different foraging areas.

The northern end of the SAB provides both an important migratory corridor and overwintering refuge for NRU and PFRU females that use productive foraging sites in the MAB. However, the southern end of the SAB, specifically the intercoastal waterways and adjacent continental shelf habitats around Cape Canaveral, FL, represents a foraging hotspot for PFRU turtles (both male and female) that use the adjacent breeding/nesting sites in east central Florida [9,22,37,40]. While a non-migratory or ‘resident’ foraging strategy might allow turtles to reallocate resources otherwise used in migration towards a greater investment in growth and reproduction, this behavior might also come at a cost if local foraging sites exhibit lower habitat quality and productivity or greater competition. Indeed, turtles (males and females) that remain resident in east central FL are smaller than migratory males [38,40] and ‘resident’ females show lower emergence success–a proxy for egg quality–than females that migrate south to the SNWA [9]. These differences, again, suggest a tradeoff between the cost of migration and the benefits of seeking out more productive foraging areas.

For nesting sites on the west coast of Florida, the prevalence of PFRU females that use the SNWA foraging area shifts to a greater contribution of females that use the EGoM foraging area, adjacent to their nesting beaches (Fig 5). Genetic subdivision between PFRU turtles nesting in southwestern vs. central western FL [59] is accompanied by subtle differences in the proportions of females originating from different foraging areas. Additionally, females from the distinct subpopulation that nest in the Dry Tortugas, FL predominantly use the SNWA foraging area, especially The Bahamas [12], while those in the Northern Gulf of Mexico Recovery Unit predominantly use the EGoM and Northern Gulf of Mexico (NGoM) foraging areas (Fig 5). Because these patterns are based satellite tracking data alone, sample sizes are relatively low and population-wide inferences are potentially less robust. As exemplified by studies in the NRU and eastern PFRU, stable isotope analysis might provide a robust and cost-effective alternative for management agencies seeking to identify and monitor the foraging-area composition of loggerheads at nesting sites in the western PFRU, DTRU, and NGMRU.

Satellite-tracking studies suggest that like the aforementioned ‘resident’ PFRU turtles that forage *and* breed in east central FL (southern SAB), but unlike most NRU females, many turtles nesting in southern Florida and the Gulf of Mexico tend to migrate relatively short distances and use nesting sites located within their foraging area. Whether a more ‘resident’ strategy of using nesting sites within the same foraging area or the alternative strategy of using nesting sites in a different foraging area (‘non-resident’) entails a reproductive advantage (or disadvantage) for turtles nesting in southern FL and the Gulf of Mexico remains unknown. Reproductive carry-over effects (e.g., clutch size, clutch frequency, and remigration interval) between ‘resident’ and ‘non-resident’ females have yet to be analyzed. However, the quality and productivity of more proximate foraging habitats might be sufficiently high to support the energy demands of reproduction such that fewer females exhibit a ‘non-resident’ strategy.

Although never the most predominant foraging area used by turtles in this study, foraging sites along and adjacent to the Yucatan Peninsula in the Southern Gulf of Mexico (SGoM) support females from three of the four loggerhead Recovery Units in the United States (Fig 5). The SGoM may very well be the predominate foraging area used by the loggerheads nesting in Mexico, including beaches in Quintana Roo, Yucatan, an important rookery for the Greater Caribbean Recovery Unit [3]. However, neither stable isotope nor satellite tracking data have been used to identify the post-nesting foraging destinations of females in this rookery or other rookeries across the Greater Caribbean. Whereas tag returns indicate that females nesting in Mexico do use foraging areas outside the SGoM [61], the “major” foraging area designated for loggerheads in the SGoM [60–63] likely supports a mixed aggregation of adult turtles that predominately nest/breed in Mexico and northwest Caribbean, as well as turtles migrating from the southern United States and northern Caribbean (i.e., Cuba and The Bahamas, including Cay Sal).

### Conservation implications

Patterns of foraging-area origins of nesting loggerheads in the NWA vary among recovery units: females in the NRU primarily use more northerly foraging areas in the MAB, while females from the three Florida recovery units (PFRU, DTRU, and NGMRU) primarily use more southerly foraging areas in the SNWA and EGoM (Figs 4 and 5). Recovery of NRU loggerheads appears to be associated with an increasing contribution of females foraging in the MAB, therefore managing anthropogenic threats to turtle survival and habitat quality in the MAB, as well as migratory corridors and overwintering sites in the SAB, remain conservation priorities for this subpopulation [3,64–65]. Similarly, the recent rebound in PFRU nesting following a period of decline in the early 2000’s [66–67] is likely linked to conditions in the SNWA and EGoM foraging areas. Managing threats and productivity in these foraging areas will continue to support the health and stability of the PFRU, a globally important subpopulation of loggerheads [3,64–65].

Identifying the primary foraging area for each recovery unit is clearly important. However, the variation among foraging-area contributions displayed across nesting sites in Figs 4 and 5 confirm two important patterns that will continue inform future interpretations and actions. First, the patterns indicate that each recovery unit is composed of females that originate from multiple foraging areas, not just one. For this reason, any attempt to evaluate region-specific threats or management strategies by assessing changes in abundance and productivity at nesting sites would be confounded without monitoring changes in the foraging-area composition of the breeding population [9,50]. Second, the patterns indicate that each foraging area is used by females from multiple recovery units. Such ‘mixed stock’ aggregations characterize the foraging areas used by both adult and juvenile loggerheads [68–70]. Conservation actions aimed to mitigate prominent threats in one foraging area would therefore enhance the viability of multiple genetic stocks and life stages. For this reason, because PFRU loggerheads use all six foraging areas in the NWA, efforts to protect this globally important subpopulation would likely include management actions and policies that encompass the foraging areas also used by loggerheads from the less prominent recovery units: NRU, DTRU, and NGMRU. Protection of such ‘umbrella’ stocks in this and other marine turtle populations will help preserve other smaller, more vulnerable stocks, including stocks that receive less protection in other areas of their geographic range (e.g., loggerheads in the Greater Caribbean Recovery Unit).

Marine turtles, like other migratory species, are difficult to access during most of their lives. Breeding sites where migratory species congregate for reproduction (e.g., nesting beaches, spawning sites, rookeries, and calving grounds) offer invaluable opportunities to conduct population assessments of these threatened species [1]. As the patterns summarized in this study show, breeding aggregations are frequently composed of individuals that use different foraging areas, each associated with a different migratory distance and route, and distinguished by its own ecological characteristics, environmental conditions, anthropogenic threats, and management actions. Understanding the foraging-area composition of breeding aggregations is therefore critical for contextualizing changes in population abundance, productivity, and stability over time, as well as evaluating population-level responses to management strategies.

## Supporting information

S1 Fig Distribution of stable isotope ratios among nesting sites. Stable carbon and nitrogen isotope (δ^13^C and δ^15^N) values for 596 yolk samples and 150 skin samples collected from female loggerheads nesting at seven sites in the Northern Recovery Unit (NRU) in 2011–2013. Different symbols indicate different nesting sites. Colored icons represent samples that were assigned to one of the three foraging areas in the Northwest Atlantic Ocean (Fig 1) with posterior probabilities ≥0.80 in a discriminant function analysis (N = 598). Fifteen individuals with assignable duplicate samples were assigned to the same foraging area in both samples. Grey icons represent 133 samples that could not be assigned to one of the three foraging areas at a posterior probability ≥0.80. *MAB*, Mid-Atlantic Bight; *SAB*, South Atlantic Bight; *SNWA*, Subtropical Northwest Atlantic.
(TIF)

S1 Table Training data. Skin stable isotope values of 60 adult loggerheads from known foraging areas (either through satellite tracking or from sample collection at a foraging site) collected between 2004 and 2011 (Pajuelo et al. 2012).
(CSV)

S2 Table Stable isotope data. Stable isotope ratios (following appropriate conversions) and posterior probabilities for foraging-area assignment of the 596 yolk samples and 150 skin samples collected from individual loggerhead turtles in this study.
(CSV)

S3 Table Literature review. Summary of published articles that use either stable isotopes or satellite tracking to identify the foraging-area origins/destinations of nesting (females) or breeding (males) loggerhead turtles in the NWA.
(CSV)

S1 File Metadata associated with S1 Table, S2 Table, and S3 Table. (PDF)
