# Supplementary figures and images for: Identifying patterns in foraging-area origins in breeding aggregations of migratory species: Loggerhead turtles in the Northwest Atlantic
Source: PLoS One. 2020 Apr 13;15(4):e0231325. doi: 10.1371/journal.pone.0231325 (PMC7153900; doi:10.1371/journal.pone.0231325)

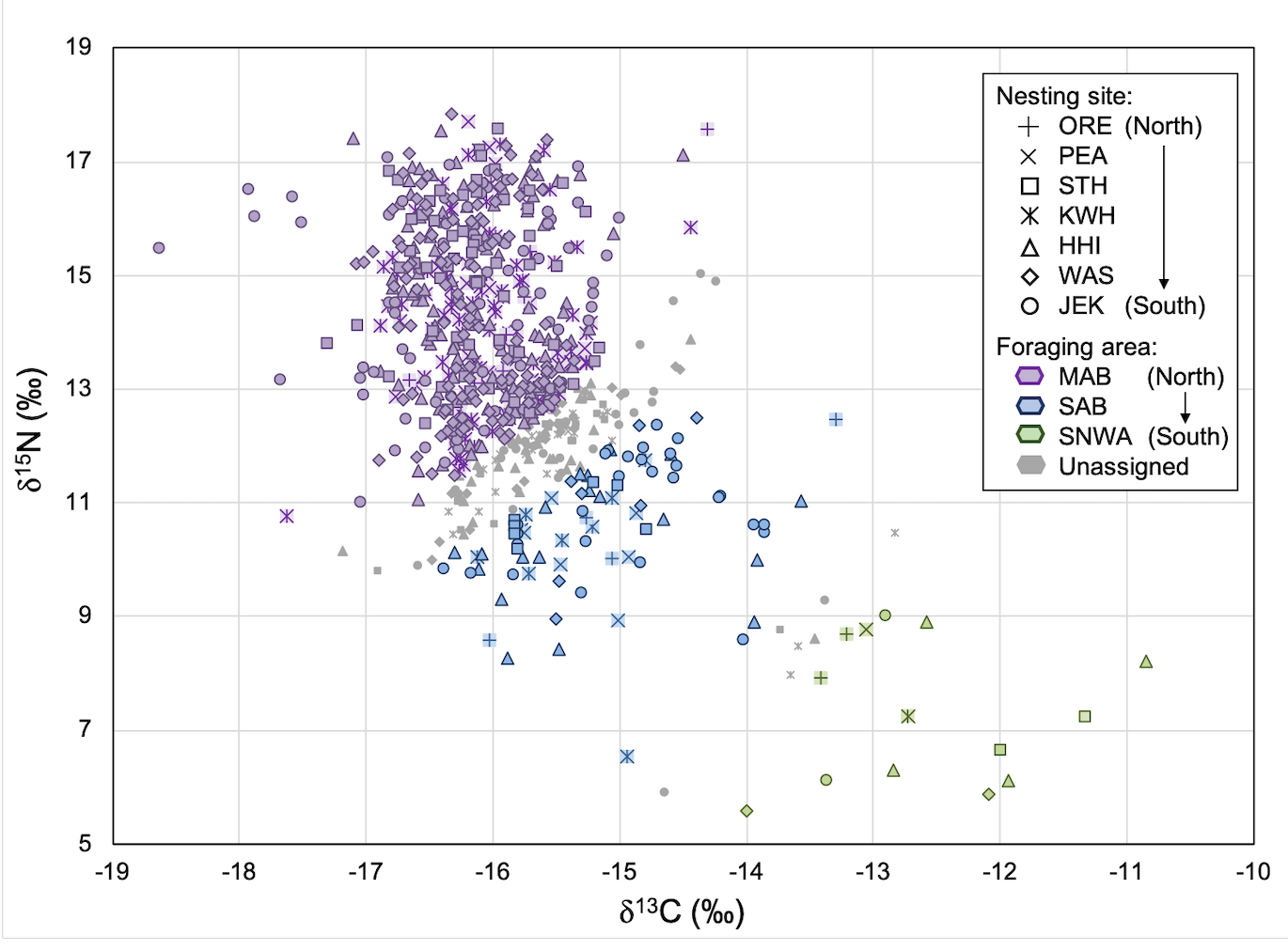

Supplement: S1 Fig — Stable carbon and nitrogen isotope (δ13C and δ15N) values for 596 yolk samples and 150 skin samples collected from female loggerheads nesting at seven sites in the Northern Recovery Unit (NRU) in 2011–2013. Different symbols indicate different nesting sites. Colored icons represent samples that were assigned to one of the three foraging areas in the Northwest Atlantic Ocean (Fig 1) with posterior probabilities ≥0.80 in a discriminant function analysis (N = 598). Fifteen individuals with assignable duplicate samples were assigned to the same foraging area in both samples. Grey icons represent 133 samples that could not be assigned to one of the three foraging areas at a posterior probability ≥0.80. MAB, Mid-Atlantic Bight; SAB, South Atlantic Bight; SNWA, Subtropical Northwest Atlantic. (TIF) [file pone.0231325.s001.tif]
