# Supplementary material for: Identifying patterns in foraging-area origins in breeding aggregations of migratory species: Loggerhead turtles in the Northwest Atlantic
Source: PLoS One. 2020 Apr 13;15(4):e0231325. doi: 10.1371/journal.pone.0231325 (PMC7153900; doi:10.1371/journal.pone.0231325)
Supplement: S1 File — (PDF) [file pone.0231325.s005.pdf]

DATA SUPPLEMENT METADATA

Identifying patterns in foraging-area origins in breeding aggregations of migratory species: loggerhead turtles in the Northwest Atlantic

Pfaller JB, Pajuelo M, Vander Zanden HB, Andrews KM, Dodd MG, Griffin DB, Godfrey MH, Ondich BL, Pate SM, Williams KL, Shamblin BM, Nairn CJ, Bolten AB, Bjorndal KA

METADATA FOR “S1 TABLE.CSV”

(COLUMN) HEADING - Explanation

(A) TURTLE – Individualized turtle number.

(B) YEAR – Collection year.

(C) LIFE STAGE – Life stage where tissue sample was collected.

FORAGING – Foraging area
FORAGING/MATING – Foraging area that also serves as a mating area
NESTING – Nesting beach

(D) STATE – State where tissue was collected.

FL – Florida
GA – Georgia
NC – North Carolina

(E) SAT TAG – Was a satellite tag used to determine foraging area?

N – No; at foraging sites
Y – Yes; at nesting sites or breeding/mating sites

(F) FORAGING – Foraging site, state, or country determined by satellite tracking or capture.

BAH – The Bahamas
DE – Delaware
FLB – Florida Bay, FL (captured in known foraging area; not satellite tracked)
FLC – Cape Canaveral, FL
FLK – Florida Keys, FL
GA – Georgia
MD – Maryland
NC – North Carolina
NJ – New Jersey
SC – South Carolina
VA – Virginia

(G) AREA – Foraging area determined by satellite tracking or capture (Fig 1)

MAB – Mid-Atlantic Bight
SAB – South Atlantic Bight
SNWA – Subtropical Northwest Atlantic

(H) d13C – Delta Carbon-13 value (‰).

(I) d15N – Delta Nitrogen-15 value (‰).

METADATA FOR "S2 TABLE.CSV"

(COLUMN) HEADING - Explanation

(A) TURTLE – Individualized turtle number; individuals sampled twice have same number.

(B) SITE – Sampling site / nesting beach.

HHI – Hilton Head Island, South Carolina (SC)
JEK – Jekyll Island, GA
KWH – Kiawah Island, SC
ORE – North of Oregon Inlet, North Carolina (NC)
PEA – Pea Island, NC
STH – South Island, SC
WAS – Wassaw Island, Georgia (GA)

(C) STATE – State of sampling site / nesting beach.

GA – Georgia
NC – North Carolina
SC – South Carolina

(D) YEAR – Collection year.

(E) DUP – Duplicate samples from same individual; blank cells indicate samples from individuals sampled only once.

DUP – Duplicate samples that were included because the other sample was unassignable or because the other sample was assignable to the same foraging area.
DUP-EX – Duplicate samples that were excluded because they were unassignable or because the other sample was assignable to the same foraging area.

(F) TYPE – Type of tissue.

SKIN – Epidermal biopsy sample collected from shoulder-region of turtles.

YOLK – Yolk sample collected from research egg; yolk values were converted to skin values using equations in Kaufman et al. (2014):

$\delta_{13}C_{skin} = 0.58 \times \delta_{13}C_{yolk} - 4.27$
$\delta_{15}N_{skin} = 1.16 \times \delta_{15}N_{yolk} -1.90$

(G) d13C – Delta Carbon-13 value (‰). If TYPE is SKIN, then no yolk-skin conversions were made. If TYPE is YOLK, then value represents yolk-to-skin converted values.

(H) d15N – Delta Nitrogen-15 value (‰). If TYPE is SKIN, then no yolk-skin conversions were made. If TYPE is YOLK, then value represents yolk-to-skin converted values.

(I) posterior.MAB – Posterior probability of assignment to the Mid-Atlantic Bight foraging area (MAB).

(J) posterior.SAB – Posterior probability of assignment to the South Atlantic Bight foraging area (SAB).

(K) posterior.SNWA – Posterior probability of assignment to the Subtropical Northwest Atlantic foraging area (SNWA).

(L) 80%ID – Assigned foraging area when posterior probability was greater than or equal to 0.80.

MAB – Mid-Atlantic Bight
SAB – South Atlantic Bight
SNWA – Subtropical Northwest Atlantic
UNK – Unknown; 0.8 threshold was not met for any of the three foraging areas

---

METADATA FOR “S3 TABLE.CSV”

(COLUMN) HEADING - Explanation

(A) METHOD – Method used to determine foraging area.

SIA – Stable isotope analysis
SAT – Satellite tracking

(B) RECOVERY UNIT – Recovery Unit of turtles as stipulated in NMFS and USFWS (2008).

DTRU – Dry Tortugas Recovery Unity
NGMRU – Northern Gulf of Mexico Recovery Unit
NRU – Northern Recovery Unit
PFRU – Peninsular Florida Recovery Unit

(C) SITE – Site where tissue sample was collected or where satellite tracking was initiated.

(D) STATE – State where tissue sample was collected or where satellite tracking was initiated.

AL – Alabama
FL – Florida
GA – Georgia
NC – North Carolina
SC – South Carolina
VA – Virginia

(E) SITE CODE – Site code in Figs 4 and 5.

(F) LIFE STAGE – Life stage of turtles that were sampled or tracked.

MATING (males) – Male turtles captured in Port Canaveral, FL and satellite tracked during post-mating migrations. Does not include four non-reproductive males.
NESTING – Females sampled on nesting beaches or tracked during post-nesting migrations.

(G) YEAR RANGE – Year range when samples were collected or when satellite tracking was conducted. When gaps in data collection were greater than two years apart at the same site, we show the data for different periods separately here but combine them in Figs 4 and 5. Data collected in same years or consecutive years are combined.

(H-M) Number of turtles assigned/tracked to different foraging areas – Foraging area assignments and satellite tracking destinations are frequently used in multiple studies and re-analyzed in different ways. We have attempted to use the most recent and/or robust analyses, while counting each individual only once (to the best of our knowledge).

EGoM – Eastern Gulf of Mexico
NGoM – Northern Gulf of Mexico
MAB – Mid-Atlantic Bight
SAB – South Atlantic Bight
SGoM – Southern Gulf of Mexico
SNWA – Subtropical Northwest Atlantic
na – Training data used to assign foraging areas via stable isotope analysis did not include individuals that migrated to these foraging areas. Therefore, individuals could not be assigned to these foraging areas in the discriminant function analyses.
** Excludes four non-reproductive males satellite tagged in Port Canaveral, FL because these turtles were not undergoing post-breeding migrations.
*** Includes one turtle that migrated to the western Gulf of Mexico (area not shown in Fig 4 and 5).

(N) Primary or consensus reference/s – Primary or consensus reference/s used to determine foraging area origins/destinations. When individuals are used in multiple studies, we have attempted to use the most recent and/or robust analyses, while counting each individual only once (to the best of our knowledge).

(O) Other reference/s – Reference/s that include all or some of the same individuals as a primary or consensus reference. These include primary references that are included in a consensus reference or secondary references that include data from a primary reference.

** Ceriani et al. (2017) assigned 749 nesting females to foraging areas in the NWA but did not provide the number of turtles that were assigned to each foraging area. Therefore, these data could not be included in Figs 4 and 5.
